# An adaptogenic role for omega-3 fatty acids in stress; a randomised placebo controlled double blind intervention study (pilot) [ISRCTN22569553]

**DOI:** 10.1186/1475-2891-3-20

**Published:** 2004-11-28

**Authors:** Joanne Bradbury, Stephen P Myers, Chris Oliver

**Affiliations:** 1 Australian Centre for Complementary Medicine, Education and Research, a joint venture between University of Queensland and Southern Cross University, PO Box 157, Lismore, NSW 2480, Australia; 2 School of Pharmacy, University of Queensland, St Lucia, QLD 4067, Australia; 3 School of Natural and Complementary Medicine, Southern Cross University, PO Box 157, Lismore NSW 2480, Australia; 4 Blackmores Research Institute, PO Box 157, Lismore NSW 2480, Australia

## Abstract

**Background:**

There is evidence for an adaptive role of the omega -3 fatty acid, docosahexaenoic acid (DHA) during stress. Mechanisms of action may involve regulation of stress mediators, such as the catecholamines and proinflammatory cytokines. Prevention of stress-induced aggression and hostility were demonstrated in a series of clinical trials. This study investigates whether perceived stress is ameliorated by DHA in stressed university staff.

**Methods:**

Subjects that scored ≥ 17 on the Perceived Stress Scale were randomised into a 6-week pilot intervention study. The diet reactive group was supplemented with 6 g of fish oil containing 1.5 g per day DHA, while the placebo group was supplemented with 6 g a day of olive oil. The groups were compared with each other and a wider cross sectional study population that did not receive either active or placebo intervention.

**Results:**

There was a significant reduction in perceived stress in both the fish oil and the placebo group from baseline. There was also a significant between-group difference between the fish oil group and the no-treatment controls in the rate of stress reduction (*p *< 0.05). However, there was not a significant between-group difference between the fish oil and the placebo group, nor the placebo group and the control group.

These results are discussed in the context of several methodological limitations. The significant stress reductions in both the fish oil and the placebo group are considered in view of statistical regression, an effect likely to have been exaggerated by the time course of the study, a large placebo effect and the possibility of an active effect from the placebo.

**Conclusion:**

There were significant differences (*p *< 0.05) in the fish oil group compared with no-treatment controls. This effect was not demonstrated in the placebo group. As a pilot study, it was not sufficiently powered to find the difference between the fish oil group and the placebo group significant. Further work needs to be undertaken to conclusively demonstrate these data trends. However, the findings from this research support the literature in finding a protective or 'adaptogenic' role for omega-3 fatty acids in stress.

## Background

Rousseau and Moreau *et al *[1] demonstrated an ameliorated cardiac response to a mild socio-social stress in DHA (the omega-3 fatty acid, docosahexaenoic acid) fed rats. The feeding schedule induced mild increases in heart rate in the sunflower oil fed group but not the DHA group. A corresponding increase in norepinephrine was significant only in the sunflower oil group. DHA also decreased systolic and diastolic blood pressure. The beneficial cardiovascular alterations, evident within a few weeks of supplementation, corresponded with high cardiac phospholipid membrane levels of DHA found on post-mortem examination.

Mills and Prkachin *et al *[2] found an effect from borage oil (found to rapidly increase membrane dGLA, the omega-6 dihomogammalinolenic acid) but not fish oil (rich in DHA) in cardiac parameters of stress reactivity in humans. Unfortunately, the potential confounds were not adequately discussed, bringing into question the reliability of these results. For instance, there was no mention of subject withdrawals or dropouts, a flush-out period, background diets, or background stress levels. Chronic stress levels have subsequently been demonstrated to influence reactivity to an acute stressor [3] and the effectiveness of DHA to reduce stress [4].

A research group in Japan have shown a protective effect of DHA during stress. A multi-centred randomised, placebo-controlled, double blind study involving 53 medical students and 3 months supplementation with 1.5 g/d was timed to coincide with a period of intense stress. They found that aggression towards others was significantly increased in the control group by 8.9% from baseline (*p *< 0.007) during the final examinations. There was no difference in aggression in the DHA group [5]. DHA prevented an increase in aggression during the examination period.

The second study was modelled on the previous study with the major difference being timing [6]. A similar but non-stressed sample 46 of university students were tested for aggression. The second study was designed to not coincide with any periods of academic stress. It commenced at the start of the summer holidays. The researchers found that DHA does not affect aggression of normal volunteers under non-stressful conditions.

Hostility was also found to increase significantly during psychological stress[7]. In a randomised, placebo-controlled, double blind study, 41 students took either 1.5 g/d DHA or placebo (soy oil) for 3 months. Hostile responses were significantly increased by from 27% (baseline) to 92% (during exams) in the control group, where there were no significant changes in the DHA group (*p *< 0.01). There were highly significant between-group differences (*p *< 0.002). The same researchers demonstrated that hostility levels significantly decreased in a population of university staff taking DHA supplementation compared with no change in hostility levels in subjects taking the placebo [7]. DHA appears to have an adaptive effect on hostility.

Sawazaki and Hamazaki *et al *[8] investigated the effect of DHA on various physiological parameters during psychological stress. Fourteen medical students took either 1.5 g/d DHA or placebo (47% olive oil, 25% rapeseed oil, 25% soy oil and 3% fish oil) for 9 weeks, culminating in a period of intense stress. While there were no significant differences between groups in epinephrine, cortisol, glucose or insulin, DHA significantly reduced plasma norepinephrine (NE) concentrations from baseline (-13%, *p *< 0.03). This reduction corresponded with a 78% increase in the ratio of epinephrine (E) to NE in the DHA group (*p *< 0.02). The higher E:NE ratios were interpreted as a favourable adaptive response to stress. This claim was substantiated by citations of studies which reported a failure to normalise the E:NE ratio during psychological stress observed in patients with duodenal ulcer. This ratio is believed to be protective as it has been associated with lower death rates in 412 older men. Thus, the authors concluded, a possible adaptive mechanism for DHA during stress may be to regulate the E:NE ratio.

Another possible mechanism whereby omega-3 fatty acids may be protective in stress is by modulation of proinflammatory cytokines. Maes and Christophe *et al *[9] found that exam stress in 27 university students significantly increased the stimulated production of many proinflammatory cytokines *ex vivo*. Subjects with low serum omega-3 fatty acid levels had significantly higher stimulated production of interleukin-6 at baseline compared with the subjects with high serum levels (*p *= 0.026) and a trend towards a significant difference during academic stress (*p *= 0.1). Stimulated production of interferon-γ and tumour necrosis factor-α was significantly greater in subjects with low serum omega-3 fatty acids (*p *= 0.02). The higher serum omega 3 levels were believed to be protective in academic stress because they were associated with lower levels of pro-inflammatory cytokines

The primary aim of the present study is to investigate whether manipulation of dietary fats has an effect on perceived stress, as measured by the Perceived Stress Scale (PSS). The hypothesis is that DHA will ameliorate stress in moderate to highly stressed university staff.

## Methods

**Figure 1:**
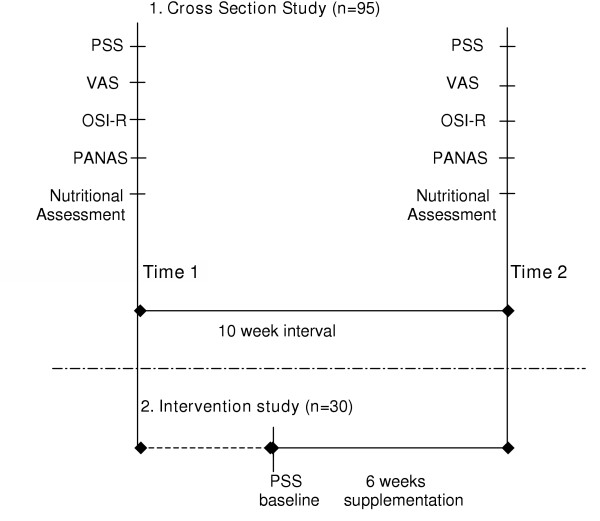
**A synoptic overview of the research design showing the smaller intervention study nested within the larger cross sectional study. **Note: PSS = Perceived Stress Study [11]; VAS = visual analogue scale; OSI-R = Occupational Stress Inventory-Revised [21]; PANAS=Positive and Negative Affect Scales [22].

A small intervention study was nested within a larger prospective cross sectional study. Figure 1 illustrates the research design. The cross sectional study compared three stress measures, and correlated the respective measures with measures of mood and dietary fats intake [10]. This was repeated after a 10-week interval.

Moderately stressed subjects were randomised into a 6-week intervention study. The nutritional intervention study was designed as a double blind randomised placebo-controlled clinical trial (pilot), involving three groups. The three groups were (1) active (1.5 g/d DHA from fish oil); (2) placebo (olive oil); and (3) control (no treatment). The supplementation period was 6-weeks.

### Study Population

All procedures and processes were subject to the prior approval of the Human Research Ethics Committee at Southern Cross University.

### Sample size

A power calculation was conducted using the variability data on the PSS [11]. The α and β values were set at 0.05 and 0.8, respectively. The resulting sample size requirement was for 50 subjects in each arm to demonstrate a change of 20%, 70 subjects to demonstrate a change of 15% and 175 subjects to demonstrate a change of 10%. For logistical reasons (the research was part of an honours project), the sample size for the study was chosen to be 15 subjects in each arm. Whilst inadequately powered, it was hoped that there were enough subjects to provide data that may indicate data trends.

#### Recruitment

All staff members of Southern Cross University were invited via intra-staff email to participate in the study on the effects of dietary fats in stress. Staff that responded to the initial recruitment email had the questionnaire personally delivered. They were instructed to complete the questionnaire and return it via internal mail. Staff members were contacted by phone and invited to participate in the nutritional intervention study if the score on the PSS was greater than or equal to 17.

#### Time course for recruitment and study

Recruitment for the intervention study commenced in June 2002 and was conducted over a 4-week period. Scores from the PSS at Time 1 formed the screening for the intervention study. The PSS was re-administered at baseline. The supplementation period commenced at the end of June and was completed by mid July 2002. Time 2 questionnaires were then sent out to all subjects that had participated at Time 1. Questionnaires were returned and scored in September 2002.

#### Inclusion/exclusion criteria

Subjects were included if they were 18–60 years, had not taken a course of fish oil in the past three months, refrained from taking other nutritional supplements and/or aspirin and from radically changing their diet for the duration of the trial, and had a normal physical examination. Subjects were excluded for medical history of coronary heart disease, any type of clotting disorder, clinically diagnosed depression, psychiatric history, diabetes mellitus, or in female subjects, pregnancy or lactation. Suitable candidates undertook a brief clinical assessment.

### Randomisation and blinding

Subjects gave informed consent for the study and were subsequently randomised into two groups. A computer program was used to generate the stratified randomisation schedule. The investigators involved with the study had no knowledge of the details or results of randomisation, the study participants had no knowledge of the group to which they had been allocated, all investigators and statisticians associated with the research were blinded regarding ongoing results.

### The Nutritional Intervention

Each capsule contains 1000 mg tuna oil, with 10 mg d-alpha-Tocopherol (vitamin E). The tuna oil was standardised to contain docosahexaenoic acid (DHA) 252 mg per 1000 mg, and eicosapentaenoic acid (EPA) 60 mg per 1000 mg oil. The placebo capsules contained 1000 mg olive oil, consisting predominantly of monounsaturated fatty acids. The placebo capsules were identical to the DHA capsules in every way, including size, shape, colour and smell.

There was a 2-week wash out period prior to the commencement of the supplementation period that applied to subjects taking any form of natural or complementary medicine. All subjects were instructed to take 3 capsules with breakfast and 3 for dinner for 6 weeks.

Compliance was measured by collecting the bottles with any remaining supplements at the completion of the study. The number of remaining supplements was divided by the total number of supplements dispensed. Less than 85% compliance resulted in the withdrawal of subject data from analysis.

### Outcome

The primary outcome was the differences between groups in changes over time in perceived stress, as measured by the Perceived Stress Scale (PSS) [11]. The PSS-10 consists of 10 questions designed to measure subjective appraisal of life stress, taking into account appraisal of the ability to cope with the stress. It has adequate reliability and validity as a stress appraisal (perceived stress) measure [12]. Scores on the PSS have been correlated with other mental and physical health outcomes [13].

### Statistical (multi-level) analysis

A two-level structure was used, where level-one units were measurement occasions, consisting of Time 1 and Time 2. Level-two units were the treatment groups, consisting of T1 =the group of subjects that did not go into the intervention study; T2 = the placebo (olive oil) group and T3 = the active (fish oil) group.

## Results

### Study population

**Figure 2:**
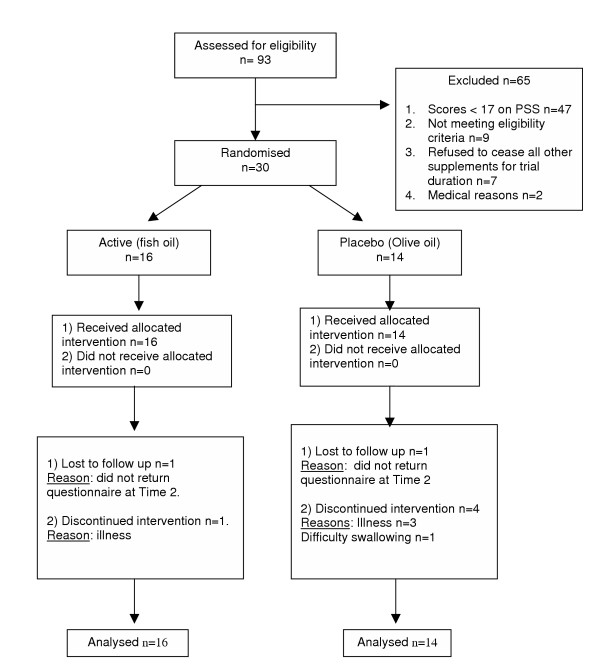
Flow of participants through each stage of the recruitment process and intervention study.

Response rate to the recruitment email was 13.6% of full-time equivalent university staff. The response rate for the return of personally delivered questionnaires was 85%. The flow of participants through the intervention study is given in figure 2. There were 47 staff members that scored ≥ 17 on the PSS. After the phone screen, 39 staff members were invited for an interview, which involved further inclusion/exclusion criteria, a clinical assessment, and obtaining informed consent.

A further 11 potential subjects were excluded as a result of the interview process; 9 because they did not want to cease other complementary medicines for the duration of the trial, 1 because of positive findings in the clinical assessment and 1 was a female subject still occasionally lactating.

### Withdrawals

A high percentage (30%) of subjects that began the intervention study were withdrawn; 3 subjects were lost to follow-up and 6 discontinued intervention; 5 due to illness related reasons and 1 because taking the supplements caused discomfort with swallowing. This resulted in 70% (n = 21) of subjects that commenced the trial participated until completion of the trial.

Multilevel analysis uniquely allows the use of unbalanced data sets. The variance component model using restricted maximum likelihood estimation was used in the hierarchical model building strategy. Absent data were assumed to be 'missing at random', implying that the reason for the missing data is not relevant to the phenomena being investigated [14].

### Randomisation

The success of randomisation was verified by dividing the subjects into groups to assess whether the distribution of group characteristics was evenly balanced. The group was divided by age, gender, BMI and gender and is given in table 1. The randomisation process resulted in an uneven distribution of group characteristics according to occupation level and gender. The numbers and proportions of the distribution of staff by occupation level are provided in table 2. The majority, 71%, of the staff randomised into the placebo group were administration staff, while the fish oil group was fairly evenly distributed. In addition, males seem to be under-represented in the placebo group.

**Table 1:** Distribution of group characteristics for subjects randomised into intervention study.

	Treatment group					
	
	Control: no-treatment		Placebo: olive oil		Active: fish oil	
	
	Mean	*SD*	Mean	*SD*	Mean	*SD*
Age	44.18	8.127	44.43	9.387	40.69	7.391
Gender	.74	.443	.79	.426	.63	.500
Body Mass Index	.00	.00	28.051	9.2384	26.684	5.8819
Occupation Status	.40	.494	.29	.469	.56	.512

Note: Dummy variables given to categorical variables: gender is coded 1 = female and 0 = males, and occupation status is coded 0 = administration staff and 1 = academic staff.

**Table 2:** Distribution of staff by occupation and gender between the three treatment groups at baseline.

	**Control**		**Placebo**		**Active**	
	No-treatment		Olive Oil		Fish Oil	
	
	n	%	n	%	n	%
**Academic**	26	40	4	29	9	56
**Administration**	37	60	10	71	7	44
Total	63	100	14	100	16	100
**Male**	17	26	3	21	6	38
**Female**	46	74	11	79	10	63
Total	63	100	14	100	16	100

Note: % = relative proportion for each subcategory (occupation and gender), n = number of subjects

These frequencies and distributions are not outside the range of expected results from the randomisation process. The main concern is that the numbers are very low in some groups. For instance, 3 male subjects in the placebo group and 4 academic staff in the placebo group may not be enough to be sensitive to significant effects, increasing the risk of a Type II error. However, the results of multivariate analysis of variance found that gender and occupational status did not have any effects on perceived stress. Therefore, the unbalanced distribution of group characteristics was not relevant.

### Preliminary and multilevel analysis

Correlations between PSS scores at Time 1 and baseline were high (0.9, *p *< 0.05) and there was no significant difference between group means on paired-samples t-tests. Therefore all subsequent analysis treated Time 1 PSS as baseline scores. Preliminary analysis involved testing for any main effects of occupation level, gender and age on the various stress measures. A two-way between-groups multivariate analysis of variance was used to test for significant interactions between the active (fish oil) and placebo (olive oil) groups over time. Age, gender and occupation levels were all added to the model with no significant effects. Therefore all models were reduced to test for the two main effects of treatment and time on perceived stress.

When the fish oil group was compared with the placebo group (olive oil), variance components analysis found no difference. No treatment effect was found but there was a trend towards an effect for the fish oil group (Wald statistic = 1.24). There was a main effect for time in both groups.

Because of the very small numbers of subjects on the study, results of the comparison between the fish oil and the placebo groups were then compared with the group of subjects that did not participate in the intervention study. Means and standard deviations for the three groups on the PSS are given in table 3. The means were much higher for subjects on the intervention study. A hierarchical model, given in table 4, investigated the effects of time, treatment and the interaction of time on treatment for the three treatment groups, where T1 = no treatment, T2 = placebo (olive oil), and T3 = active (fish oil).

**Table 3:** Means and standard deviations for the three groups on the PSS

	**No-treatment**	**Placebo (olive oil)**	**Active (fish oil)**
	Mean (*SD*)	Mean (*SD*)	Mean (*SD*)
*n*	63	14	16
**PSS**	15.83 (4.79)	23.93 (2.62)	23.94 (4.33)

Note: *n *= number of subjects

**Table 4:** Parameter estimates for models of variance components, time, treatment (T1 and T2) and interaction (of time on treatment) main effects on the Perceived Stress Scale (PSS) group means.

	**Variance Components**	**Time Main Effects**	**Treatment Main Effects**	**Interaction Main Effects**
**Fixed effects**	Coeff. (S.E.)	Coeff. (S.E.)	Coeff. (S.E.)	Coeff. (S.E.)
**β Constant**	17.639 (0.533)	18.444 (0.597)	16.479 (0.598)	15.836 (0.614)
**Time**		-1.999 (0.669)*	-2.223 (0.665)*	-0.500 (0.770)
**T2**			6.958 (1.241)*	8.092 (1.441)*
**T3**			5.372 (1.164)*	8.101 (1.365)*
**T2 × time**				-2.786 (1.679)
**T3 × time**				-6.059 (1.554)*
**Random effects**				
**Subject level residual variance**	15.498 (4.260)	16.639 (4.200)	8.226 (3.063)	9.867 (2.956)
**Time level residual variance**	18.496 (3.115)	16.599 (2.801)	16.721 (2.801)	13.930 (2.334)
**-2 log likelihood**	1020.264	1011.772	974.964	960.204

* Denotes significance (Wald statistic ≥ 1.96, where *p *≤ 0.05). Note: T2 = olive oil group, T3 = fish oil group, Coeff. = coefficient, S.E. = standard error, -2 log likelihood statistic = -2LL.

**Figure 3:**
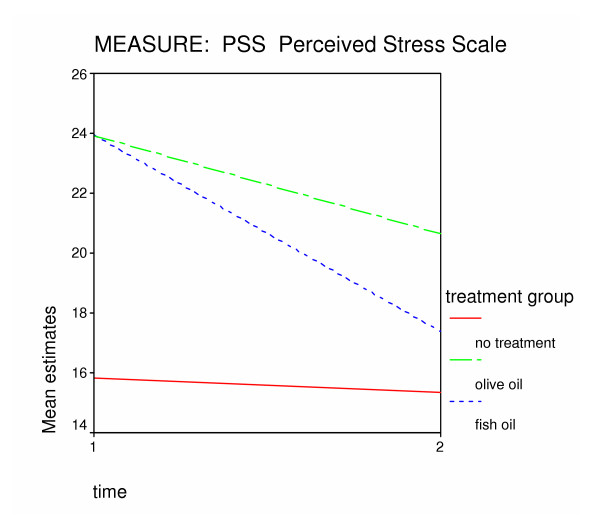
Changes over time in predicted means on the Perceived Stress Scale (PSS) for the three treatment groups.

The effects of time were significantly greater for subjects on the intervention study. There was a significant effect of treatment in the respective active and the placebo groups. Once the effects of time were accounted for, only the fish oil group estimated means were significantly different. The changes in the group means over time are provided in table 5 and illustrated in figure 3. There were substantial changes on the PSS for the two groups in the intervention study over time, but the changes in the no-treatment group were not different over time.

**Table 5:** Predicted changes over time in estimates of Perceived Stress Scale (PSS) means for each treatment group, 95% confidence intervals, and chi square value.

	**Control**	**Placebo**	**Fish oil**
Estimate	-0.500	-3.285	-6.559
95% CI sep	1.509	2.923	2.646
95% CI joint	2.152	4.170	3.755
Chi square	0.421	4.852*	23.590*

* significance determined by chi sq ≥ 3.84, the critical value of chi square for *df *= 1 when p ≤0.05. Note: 95% CI sep = 95% confidence interval considering each item separately, 95% CI joint = 95% confidence interval, considering the estimates of the three treatment groups collectively, *df *= degrees of freedom

The change over time in PSS means were not the same for the two groups in the intervention study. Inclusion in the fish oil group predicted a larger reduction in estimates of perceived stress than the placebo group. Estimated means at Time 2 on the PSS were 17.4 for the fish oil group, where those for the placebo group were 20.6.

The within-group changes over time are given in table 6. The changes over time were significant for both the placebo and fish oil groups but not the no-treatment group. The difference between the groups in the changes over time are given in table 7. The differences between the fish oil group and the no-treatment group is the only between-group difference to reach statistical significance (*p *< 0.05). The change over time in the placebo group was not significantly different from those in the no-treatment group. Changes in the placebo group over time were also not significantly different from changes in the fish oil group over time.

**Table 6:** Predicted means and 95% confidence intervals of the Perceived Stress Scale (PSS) for the treatment groups over time.

	**Control**	**Placebo**	**Fish oil**
**Time 1**			
Mean	15.836	23.929	23.938
95% CI sep	1.203	2.555	2.390
95% CI joint	1.716	3.645	3.409
**Time 2**			
Mean	15.337	20.643*	17.379*
95% CI sep	1.416	2.725	2.455
95% CI joint	2.021	3.888	3.502

Note: 95% CI sep = 95% confidence interval considering each item separately, 95% CI joint = 95% confidence interval, considering the estimates of the three treatment groups collectively. *denotes significant difference from baseline (*p *< 0.05).

**Table 7:** Estimated between-group differences in changes over time for predicted means on the Perceived Stress Scale (PSS), with confidence intervals and chi square statistic.

	**Placebo vs Control**	**Fish oil vs Control**	**Fish oil vs Placebo**
Estimate	2.786	6.059	3.273
95% CI sep	3.289	3.046	3.943
95% CI joint	4.692	4.345	5.625
Chi square	2.754	15.193*	2.647

* Significance determined by observed value of chi sq ≥ 3.84, the critical value of chi square for *df *= 1 when *p *≤ 0.05 Note: 95% CI sep = 95% confidence interval considering each item separately, 95% CI joint = 95% confidence interval, considering the estimates of the three treatment groups collectively, chi sq = chi square, vs = versus, the difference between groups

## Discussion

There were significant reductions in stress for both the fish oil and the placebo (olive oil) groups from baseline (both *p *< 0.05). The stress reduction for the fish oil group was significantly different from the no-treatment controls (*p *< 0.05). The stress reduction in the placebo group was not significantly different from the no-treatment controls. The fish oil group had more substantial stress reductions than the olive oil group, but the differences between the fish oil and the placebo groups did not reach statistical significance.

All subjects taking the nutritional intervention reported significantly less perceived stress at Time 2 (*p *< 0.05). Arguably, the key factor influencing these results is 'regression to the mean', an effect that may have been exaggerated by methodological limitations such as timing, a large placebo effect, and the possibility that the placebo was not a true placebo. These issues are discussed in the following sections.

### Statistical regression

Perhaps staff applied and were selected for the intervention study at a time when their stress levels were peaking. If this were the case, 'regression to the mean' predicts that stress levels would decrease. Factors which would exaggerate this effect are the difference in the means between the groups on the intervention study and the no-treatment controls, and the study time course corresponding with the mid-year break.

Because only 30 out of a possible 47 candidates were randomised into the intervention study, the 17 potential candidates remaining in the group receiving no intervention were thought to increase the mean of the no-treatment group. That is, 37% of the no-treatment group had high scores on the PSS (≥ 17). Therefore, this group was considered a potential control group for the intervention study.

The time course of the study may have noticeably influenced the regression to the mean. The academic year at Southern Cross University consists of two semesters. This study commenced mid-way through the first semester (Time 1). Questionnaires were reissued at the beginning of the second semester (Time 2), immediately after the mid-year break. Staff may generally have been more relaxed and less stressed after the break. The variation of stress levels associated with time may have been minimised if Time 2 had been at mid-semester 2, a time that corresponds more closely with Time 1.

Evidence of a treatment effect beyond statistical regression will be demonstrated in the between-group differences. The only such difference was between the fish oil group and the no-treatment controls. This evidence supports the hypothesis that fish oil ameliorates chronic stress.

### Methodological limitations

The present study has several important limitations, including (1) selection bias (2) unsuccessful blinding (3) inadequate power and (4) the possibility of an active effect from the 'placebo'.

#### Sampling bias

Invariably, recruitment involving self-selection entails some degree of selection bias [15]. In this instance, the advertisement was aimed at university staff interested in stress research involving the beneficial omega-3 fatty acids. Staff may have excluded themselves for weight-watching reasons, or included themselves because they were interested in the intervention. Perhaps very stressed and/or busy staff did not apply, or perhaps staff that were not stressed did not apply. Because the study ran into the mid year break, perhaps staff did not apply because of issues relating to availability during the break.

#### Power

While the results demonstrated a strong trend towards a difference between the fish oil and the olive oil groups, this difference failed to reach statistical significance with the current sample size. Post-hoc power calculations were conducted using the PSS means and standard deviations. The study had 90% power to find a 20% difference between the fish oil and the placebo groups, and only 40% power to find a difference of 10% significant. The study was underpowered to find the difference between the groups statistically significant.

#### Blinding

Although the capsules appeared in every way identical, there was one distinct difference. The fish oil capsules were often accompanied by very mild gastrointestinal disturbances in the form of a slight after taste in the mouth, which was unmistakably fishy. All the subjects taking the fish oil suspected as much because they had the taste of fish in their mouth after ingestion of the capsules.

Interestingly, half (50%) the subjects taking the placebo during this study also believed they were taking the active because they 'felt better'. The placebo group did actually report significantly less stress levels. This effect may be due regression to the mean, as previously discussed. Other factors that must be considered are the 'placebo effect' and the possibility of an active effect from the placebos.

#### The placebo effect

Half (50%) of the subjects taking the placebo believed themselves to be taking the active. If these subjects believed in the treatment and expected to benefit from it, then it is likely they reported the improvements, which may have influenced the mean of the group. If half the group were reporting an exaggerated stress reduction, then the mean of the group would show a trend towards a treatment effect. Indeed, this was observed: The placebo group demonstrated significant stress reduction; however, the change over time was not significantly different from reductions observed in the no-treatment controls.

Perhaps the most unbiased way to estimate true placebo effects is to observe the difference between a placebo group and a group of no-treatment controls in a three-arm clinical study [16]. In the present study, the change over time for the placebo group, like the fish oil group, was significant (*p *< 0.05), where the change over time for the no-treatment controls was not significant. This apparent placebo effect, however, may have been inflated by statistical regression or an active effect from the placebo.

#### The placebo

The use of olive oil as a placebo is not uncommon in essential fatty acid research [17]. However, olive oil may not be an inert substance in brain lipid chemistry. Oleic acid, the major lipid in olive oil, is related to an endogenous sleep-inducing substance. Isolation of a chemical from the cerebrospinal fluid of sleep deprived cats, led to its identification as *cis*-9,10-octadecenoamide (oleamide), an analogue of 9-octadecenoic acid (oleic acid) [18]. Oleamide, but not oleic acid, was found to improve the action of serotonin (5-hydroxytryptamine), which implies a role for this molecule in mood, alertness and sleep [19]. Cell membranes have been shown to catalyse the synthesis of oleamide from oleic acid [20]. In addition, the rat brain demonstrated control of increased levels of *cis*-9,10-octadecenoamide (oleamide) by conversion into oleic acid [18]. Understanding of the lipid chemistry involved with the neuromodulation of sleep and mood is still incomplete. However, the assumption that oleic acid is neutral in lipid neurochemistry is questionable.

The issue of a correct choice of placebo for essential fatty acid research is difficult. Most studies use omega-6 rich oil, such as corn oil, soy oil, or safflower seed oil. However, this practice is not completely unbiased as the omega-6 oil competes with omega-3 fatty acids for metabolism. Given in high enough doses, the omega-6 oils will overwhelm delta-6 desaturase and inhibit the metabolism of the omega-3 fatty acids.

This methodology results in a systematic measurement error, where the omega-3 metabolism in the placebo group may be suppressed. Further, the placebo is not a true placebo if it has a specific effect on the measurement outcome. Between-group differences in these studies may be enhanced. Alternatively, soy oil has been shown to increase omega-3 levels in omega-3 deficiency. As the regulation of the end products of n-3:n-6 blood levels is complex and not fully understood, it is probably best to avoid using these fatty acids as a placebo. This practice may increase the variance in the measurement error to an unknown extent. The monounsaturated fatty acid found in olive oil was chosen here to avoid further imbalances between the essential fatty acids.

## Conclusions

Perceived stress was significantly reduced from baseline after 6 weeks supplementation with 1.5 g/d DHA from fish oil (*p *< 0.05). Furthermore, the difference was significant compared with no-treatment controls (*p *< 0.05). The placebo group also demonstrated significant reductions in perceived stress compared to baseline levels (*p *< 0.05). However, when compared with the no-treatment control group, the differences in perceived stress were not significant for the placebo group.

The study may have demonstrated an exaggerated regression to the mean due to its timing, a strong placebo effect or the placebo itself may have had an active effect. The question that olive oil may have a subtle but protective effect in stress has nevertheless been raised. Perhaps 6 g per day of olive oil was not sufficient dietary intake to find a difference between the placebo and no-treatment controls significant, especially with such a small sample.

This research has provided preliminary findings suggesting that DHA ameliorates perceived stress. Future research is required to conclusively substantiate the ameliorating effects of DHA in stress, and further investigate the role of olive oil and/or other dietary fats in stress reduction.

## Competing interests

This research was purely academic research and has no commercial interest. However, Blackmores Ltd, a company that sells fish oil capsules, also employs one of the authors, Chris Oliver.
